# Identification of Some New Triply Periodic Mesophases from Molten Block Copolymers

**DOI:** 10.3390/polym11061081

**Published:** 2019-06-25

**Authors:** Junhan Cho

**Affiliations:** Department of Polymer Science & Engineering and Center for Photofunctional Energy Materials, Dankook University, 152 Jukjeon-ro, Suji-gu, Yongin-si, Gyeonggi-do 16890, Korea; jhcho@dankook.ac.kr; Tel.: +82-31-8005-3586

**Keywords:** SCFT, block copolymers, new mesophases

## Abstract

Using field-theoretic simulations based on a self-consistent field theory (SCFT) with or without finite compressibility, nanoscale mesophase formation in molten linear AB and ABC block copolymers is investigated in search of candidates for new and useful nanomaterials. At selected compositions and segregation strengths, the copolymers are shown to evolve into some new nanostructures with either unusual crystal symmetry or a peculiar morphology. There exists a holey layered morphology with Im3 symmetry, which lacks one mirror reflection compared with Im3m symmetry. Also, a peculiar cubic bicontinuous morphology, whose channels are connected with tetrapod units, is found to have Pn3m symmetry. It is shown that there is another network morphology with tripod connections, which reveals P432 symmetry. The optimized free energies of these new mesophases and their relative stability are discussed in comparison with those of double gyroids and double diamonds.

## 1. Introduction

Soft matters or complex fluids have drawn tremendous attention from condensed matter researchers because of some useful features such as complexity in their various forms or structures and flexibility in changing their physical properties through simple chemical modification [1]. Block copolymers, or polymeric surfactants, are one particular soft matter that is produced by covalently joining two or more different homopolymers. In many cases, disparity in their own cohesive energies leads them to nanoscale self-assembly behaviors [2,3,4,5,6,7,8].

The simplest molten AB diblock copolymers have exhibited 1-dimensional lamellar layers, 2-dimensional hexagonal cylinders with P6/mm, body-centered cubic (BCC) spheres with Im3m symmetry, double gyroids belonging to Ia3d space group, and an orthorhombic Fddd network morphology [9,10,11]. There is a double diamond morphology belonging to Pn3m group as a metastable one [12]. Molten ABC triblock copolymers with one additional component give more diversified morphologies due to the increase in the degree of freedom [9,13,14,15,16]. There are the core-shell-type, perforated or decorated versions of the morphologies just mentioned [9,13,14,15,17], alternating I4_1_32 single gyroids [9,15,18], the centered rectangular C2mm cylinders [16], and a cubic bicontinuous P23 phase [16]. However, the morphologies reported so far are still limited for block copolymer systems. It seems that there are still many morphologies unexplored in block copolymers, as we recognize the emergence of the discontinuous mesophases with Pm3n (A15) and P4_2_/mnm (Frank–Kasper σ) symmetry in some copolymers via structural variations [19,20,21], and also the holey bicontinuous mesophases possessing I43d and Ia3d symmetry with high genera [22]. 

While in block copolymer surfactants only the handful of morphologies have ever been observed, it is seen in the compendium of hard matters or rocks and minerals that there are 230 space groups [23] and possible real-space morphologies for each group are supposed to be numerous. The objective of this study is to unveil some of hidden morphologies in the molten AB and ABC copolymers over indwelling barriers. Those morphologies studied here are peculiar with various crystal symmetries such as Im3, Pn3m, and P432. Avoiding the lengthy steps from the synthesis of desired copolymers to structure characterization, we employ a field-theoretic simulation method based on a self-consistent field theory (SCFT) for copolymer systems with [24,25] or without finite compressibility [24,26,27,28]. 

## 2. Theoretical Methods 

Consider that there are *n* chains of *N*-mers of a linear AB or ABC block copolymer melt in the canonical ensemble of volume *V*, where each chain comprises $N=N_{A}+N_{B}$ or $N=N_{A}+N_{B}+N_{C}$ monomers having an identical diameter σ and its volume $v*=\pi \sigma^{3}/6$. The hard-core volume fraction of j-monomers can then be defined as $\varphi_{j}=N_{j}/N$. The packing density of j-monomers at a local position $\vec{r}$ is described by an operator ${\hat{\eta }}_{j}(\vec{r})$, which is given by ${\hat{\eta }}_{j}(\vec{r})$ $=v*/V\cdot \sum_{i=1}^{n}\int_{\tau_{j}^{s}}^{\tau_{j}^{f}}d\tau \delta (\vec{r}-{\vec{r}}_{i}(\tau ))$, where $\delta (\vec{r})$ is Dirac’s delta function and ${\vec{r}}_{i}(\tau )$ denotes the position vector of each j-monomer on an *i*th chain through parameterization by a contour variable *τ*. The first and final j-monomers are represented by the symbols $\tau_{j}^{s}$ and $\tau_{j}^{f}$, respectively. A field variable $\eta_{j}(\vec{r})$ is then assigned to each operator ${\hat{\eta }}_{j}(\vec{r})$ in order to describe the j-monomer density at $\vec{r}$ over the entire system. The overall density ($=\sum \eta_{j}(\vec{r})$) is denoted by $\eta (\vec{r})$, and a local volume fraction $\varphi_{j}(\vec{r})$ is defined as $\varphi_{j}(\vec{r})\equiv \eta_{j}(\vec{r})/\eta (\vec{r})$. In the incompressible extreme, monomers are uniform polyhedrons and completely fill the system volume. Then, $\eta (\vec{r})\to 1$ and $\eta_{j}(\vec{r})$ becomes identical to $\phi_{j}(\vec{r})$. 

In the Edwards’ Gaussian random-walk approach [24,29], the Hamiltonian *H* for the given system is given as *H* = *H*_0_ + *W*, where *H*_0_ is given by Weiner measure of Gaussian chains as follows:

(1) $$\beta H_{0}=H_{0}/kT=\frac{3}{2\sigma^{2}}\sum_{i=1}^{n}\int_{0}^{N}d\tau {\left|\frac{d{\vec{r}}_{i}}{d\tau }\right|}^{2}$$

and *W* implies perturbing inter-monomer interactions. After taking the Hubbard–Stratonovich transformation [30], the partition function *Z* is then written as:

(2) $$Z=Z_{0}\cdot {\int \prod_{j}D\eta_{j}D\omega_{j}\left[\frac{1}{V}\int d\vec{r}\cdot q(i\omega_{A},i\omega_{B},i\omega_{C})\right]}^{n}\cdot e^{-\beta W+i\int d\vec{r}\frac{1}{v*}\omega_{j}\cdot \eta_{j}}$$

where $Z_{0}$ is the partition function of the Gaussian chains free from any segmental interactions. It is seen that the Hamiltonian *H* first based on particle description is converted to one based on field description. This procedure naturally gives a hypothetical external potential $\omega_{j}(\vec{r})$ for j-species, in which the effect of *W* on chain conformations is transferred to the partition function. The symbol *q* in Equation (2) denotes the end-segment probability density function of the Gaussian chains along the chain contour with τ, which covers from 0 to *N* or whose scaled version *s* (≡τ/N) spans from 0 to 1. 

In the imaginary external potential $i\cdot \omega (\vec{r})$, *q* satisfies a modified diffusion equation as:

(3) $$\frac{1}{N}\frac{\partial q}{\partial s}=\frac{\sigma^{2}}{6}\nabla^{2}q-i\cdot \omega \cdot q$$

along with $q(\vec{r},0)=1$. In Equation (3), *ω* takes *ω_A_*, *ω_B_*, and *ω_C_* in turn as the contour variable *s* passes through A, B, and then C blocks. It needs to be recalled that there is another function *q*^+^ conjugate to *q*, so that *q*^+^ starts reversely from the other chain end with $q^{+}(\vec{r},1)=1$. 

Regarding *W*, we take two different approaches. Firstly, a conventional treatment of *W* is taken on the basis of incompressible picture with the incompressibility constraint ($\sum \varphi_{j}(\vec{r})=1$). A phenomenological Flory–Huggins interaction parameter *χ*, which comes from the dimensionless exchange energy between ij-pairs, solely describes *W* as:

(4) $$\beta W\{{\vec{r}}_{j}\}=\sum_{i>j}\frac{nN\chi_{ij}}{V}\int d\vec{r}\varphi_{i}(\vec{r})\varphi_{j}(\vec{r})$$

An additional field $\xi (\vec{r})$ below as a Lagrange multiplier is necessary to guarantee the incompressibility condition. The partition function *Z* is analyzed in the mean-field level. The essential procedure is to get the saddle point Z* of the partition function *Z*, which requires a set of the following self-consistent field equations:

(5) $$-\sum_{i\neq j}N\chi_{ij}\varphi_{i}+i\omega_{j}+\xi (\vec{r})=0$$

(6) $$\phi_{j}(\vec{r})=\frac{1}{\frac{1}{V}\int d\vec{r}q(\vec{r},s=1)}\cdot \int_{s_{j}^{s}}^{s_{j}^{f}}ds\cdot q(\vec{r},s)q^{+}(\vec{r},1-s)$$

where $s_{j}^{s}$ ($=\tau_{j}^{s}/N$) and $s_{j}^{f}$ ($=\tau_{j}^{f}/N$) are the rescaled contour variables at start and in the end for j-monomers, respectively. Equations (3), (5), and (6) for all j-constituents need to be solved in the incompressible version of SCFT for the block copolymers. The Helmholtz free energy is then given as βA≈−lnZ* [24,26,27,28]. 

In our second approach, a finite compressibility is allowed by taking the continuum description of a copolymer system. Then, a proper equation of state (EOS) is required [25]. For such purpose, a perturbed hard sphere chain model suggested by the present author and Sanchez (Cho–Sanchez) is chosen [31]. The free energy is given as $A=A_{id}+A_{EV}+A_{nb}$, where $\beta A_{id}=-\ln Z_{0}=n\cdot \ln\eta +\mathrm{constant}$ for the Gaussian chains. The remaining terms, $A_{EV}$ and $A_{nb}$, respectively give the perturbation contribution by excluded volume (EV) to EOS [32] and a Bethe–Peierls-type mean-field energy between nonbonded monomers as [33]:

(7a) $$\frac{\beta A_{EV}}{nN}=\left\{\frac{3}{2}\left[\frac{1}{{\left(1-\eta \right)}^{2}}-\left(1-\frac{1}{N}\right)\frac{1}{1-\eta }\right]-\frac{1}{N}\left[\ln\left(1-\eta \right)+\frac{3}{2}\right]\right\}$$

(7b) $$\frac{\beta A_{nb}}{nN}=\frac{1}{2}\cdot \beta \cdot \sum_{ij}\phi_{i}\phi_{j}{\bar{\varepsilon }}_{ij}\cdot u(\eta )$$

where ${\bar{\varepsilon }}_{ij}$ implies the characteristic i,j-contact interactions. The u(η) represents the density dependence of the Bethe-Peierls energy as $u(\eta )=f_{p}\cdot [{\left(\gamma /C\right)}^{p/3}\eta^{p/3}-{\left(\gamma /C\right)}^{2}\eta^{2}]$, where γ and *C* are respectively $1/\sqrt{2}$ and π/6. For the widely used Lennard–Jones potential, *p* and *f_p_* are 12 and 4, respectively. This Bethe–Peierls mean-field energy is an improvement over Bragg–Williams (van der Waals) mean-field energy by taking the local packing of nearest neighbors into account [33]. The Cho–Sanchez model necessitates three homopolymer parameters such as ${\bar{\varepsilon }}_{jj}$ for self-interactions, $\sigma_{j}$, and $N_{j}$. It is well known that a homopolymer with larger ${\bar{\varepsilon }}_{jj}$ is denser and less compressible than one with smaller ${\bar{\varepsilon }}_{jj}$. There is an additional parameter ${\bar{\varepsilon }}_{ij}$ for cross i,j-interactions to describe mixture phase behaviors. Using the given EOS model, it was suggested that $\beta W\{{\vec{r}}_{j}\}=\int d\vec{r}\beta f^{ni}({\hat{\eta }}_{j}(\vec{r}))$, where $f^{ni}({\hat{\eta }}_{j}(\vec{r}))$ is the localized non-Gaussian free energy ($f^{ni}$ $\equiv \left(A_{EV}+A_{nb}\right)/V$) per unit volume [25].

The partition function *Z* in Equation (2) is to be analyzed again in the mean-field level. The resultant set of self-consistent field equations are given below in case of the compressible system:

(8) $$-Nv*\cdot \frac{\partial \beta f^{ni}}{\partial \eta_{j}(\vec{r})}+N\cdot i\omega_{j}=0$$

(9) $$\eta_{j}(\vec{r})=\frac{\eta }{\frac{1}{V}\int d\vec{r}q(\vec{r},s=1)}\cdot \int_{s_{j}^{s}}^{s_{j}^{f}}ds\cdot q(\vec{r},s)q^{+}(\vec{r},1-s)$$

It should be noted that there is no need for a Lagrange multiplier to suppress compressibility. Solving Equation (3) and (7–9) for all j-constituents fulfills SCFT based on the suggested Edwards Hamiltonian for the compressible block copolymers. The bulk density *η* of the disordered copolymer at a given T and P come into play in order to describe pressure effects. 

The segregation strengths in the compressible approach can be monitored through a proper effective Flory–Huggins *χ* that carries its dependence not only on temperature but also on pressure. It has been suggested in our previous works that *χ* consists of two terms as $\chi =\chi_{H}+\chi_{S}$ [34,35]. The former $\chi_{H}$ (=$\beta \Delta \bar{\varepsilon }\cdot \left|u(\eta )\right|/2$) indicates the conventional enthalpic term with density dependence, where $\Delta \bar{\varepsilon }$ is the exchange energy between ${\bar{\varepsilon }}_{ij}$’s as $\Delta \bar{\varepsilon }\equiv {\bar{\varepsilon }}_{ii}+{\bar{\varepsilon }}_{jj}-2{\bar{\varepsilon }}_{ij}$. The latter $\chi_{S}$ (=$P_{\varphi }^{2}v*/2kT\eta B_{T}$) indicates the entropic term, where $P_{\phi }^{}$ is the composition coefficient of pressure as $P_{\phi }^{}$ $\equiv {\partial P/\partial \phi )}_{T,v}$ and $B_{T}$ is the bulk modulus as $B_{T}$ $\equiv \eta \cdot \partial P/\partial \eta {)}_{T,\phi ’s}$. $P_{\phi }^{}$ gives the compressibility difference between components as $P_{\varphi }\propto ({\bar{\varepsilon }}_{ii}-{\bar{\varepsilon }}_{jj})$ at ϕ→1/2. It needs to be mentioned that the given notion of the effective χ is perfectly harmonious with that from Sanchez–Lacombe theory [36,37,38].

## 3. Results and Discussion

### 3.1. Im3

Let us first discuss a morphology with crystal symmetry Im3. The discovery of this nanostructure was fortuitous. In our previous work on the pressure effects on morphology development in compressible ABC linear triblock copolymer melts, we took a model system from the copolymer having the block sizes with $\left(N_{A},N_{B},N_{C})=(110,55,55\right)$, whose homopolymer molecular parameters are listed in Table 1. With such block sizes, A/B or A/C pair prefers curved micelles, while A/(B+C) pair or B/C pair favors flat micelles. It is seen that $\varepsilon_{AA}>\varepsilon_{BB}=\varepsilon_{CC}$, which implies that A is denser than either B or C, and the densities of B and C are identical. To characterize the copolymer, the cross interaction parameters, $\varepsilon_{AB},\;\varepsilon_{AC}$, and $\varepsilon_{BC}$ were required. We choose ${\bar{\varepsilon }}_{AB}/{\left({\bar{\varepsilon }}_{AA}{\bar{\varepsilon }}_{BB}\right)}^{1/2}$ = 1.0087, ${\bar{\varepsilon }}_{AC}/{\left({\bar{\varepsilon }}_{AA}{\bar{\varepsilon }}_{CC}\right)}^{1/2}$ = 1.0088, and ${\bar{\varepsilon }}_{BC}/{\left({\bar{\varepsilon }}_{BB}{\bar{\varepsilon }}_{CC}\right)}^{1/2}$ = 0.9880 to yield $N\chi_{AB},\;N\chi_{AC}$, and $N\chi_{BC}$ = 16.30, 16.13, and 16.77, respectively, at ambient pressure and at 400 K. It was revealed that C2mm cylinders are competing with lamellae, hexagonal P6/mm cylinders, *Im3m* BCC spheres, and the mesophase of lower *P23* symmetry [16].

**Figure 1:**
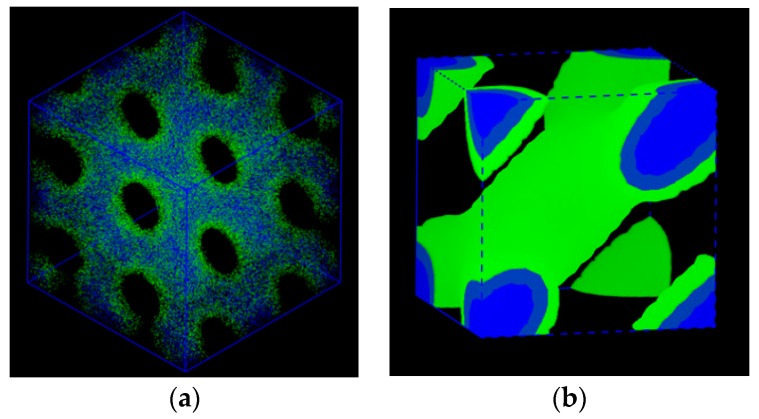
Three-dimensional morphology of the evolved nanostructure from compressible ABC copolymer melt (**a**) in the simulation box of 12 × 12 × 12$R_{G}^{3}$ and (**b**) in the unit cell of 6 × 6 × 6$R_{G}^{3}$ (1/8th of the simulation box). B and C domains are represented by green and blue color, respectively, and A domain as the matrix is erased intentionally.

Starting with the pre-determined BCC spheres, the SCFT simulations for the copolymer melts were undertaken in a periodic cubic box of 12 × 12 × 12$R_{G}^{3}$, where $R_{G}$ was the copolymer gyration radius as $R_{G}^{}=\sigma \cdot {\left(N/6\right)}^{1/2}$. The simulation box was discretized into 32^3^ lattice cells, and each chain contour was discretized into $N_{A}+N_{B}+N_{C}$ segments for A, B, and C blocks, respectively. The modified diffusion equation in Equation (3) along with Equations (7)–(9) were solved via the pseudospectral scheme [39]. The iteration at a given condition is continued until the variation of $\omega_{j}$’s is less than ~2.5 × 10^−6^. Using the single core of the Intel Xeon processor, it takes ~24 min per 1k iterations for the copolymer melts, and 40k iterations are necessary to reach the target tolerance. In the early stage, BCC stays, but eventually at the present compositions, there evolved a totally different and new morphology. Using Biovia Material Studio Mesodyn package, we visualized the 3-dimensional morphology of the copolymer melt, which is depicted in Figure 1. It is seen that the unit cell of the structure seems only 6 × 6 × 6$R_{G}^{3}$ just as that of BCC. In our first look at the morphology, it possesses holey layers, not the dispersed micellar spheres. 

**Figure 2:**
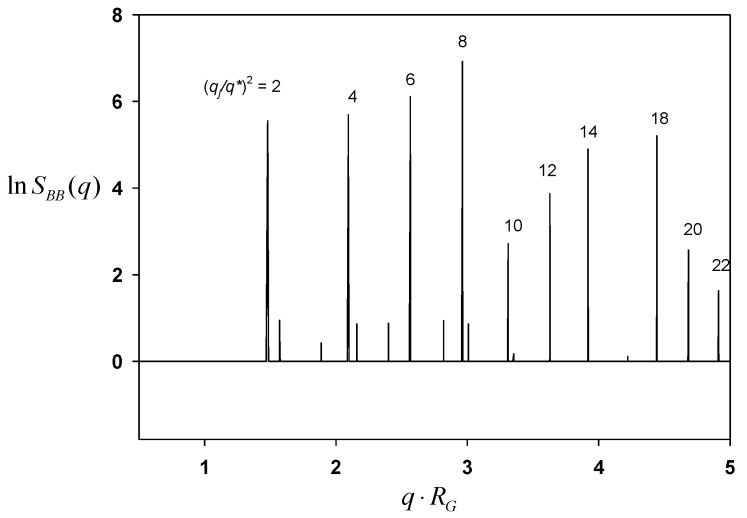
Correlation function $S_{BB}(q)$ for B block plotted against the scattering vector *q* for the morphology given in Figure 1.

The symmetry of the newly obtained morphology can be probed through a Fourier transformed second-order correlation function $S_{jj}(q)$ as:

(10) $$S_{jj}(q)=\int_{}^{}d\vec{r}\cdot e^{-i\bar{r}\cdot \vec{q}}\cdot \langle \left(\eta_{j}(\vec{r})-\eta_{j})(\eta_{j}(0)-\eta_{j}\right)\rangle$$

where $\vec{q}$ implies the scattering vector and $q=\left|\vec{q}\right|$. Figure 2 displays $S_{BB}(q)$ for B block as a function of the dimensionless $q\cdot R_{G}$. There are a series of characteristic peaks at $q\cdot R_{G}$ = 1.4810, 2.0949, 2.5651, 2.9619, 3.3115, 3.6276, etc. The calculation of $2\cdot {\left(q/q_{1}\right)}^{2}$, where $q_{1}\cdot R_{G}$ = 1.4810 is the first one, turns all into even integers as 2, 4, 6, 8, 10, 12, etc. These numbers indicate $h^{2}+k^{2}+l^{2}$ out of (*hkl*) Miller planes. This particular series of (*hkl*) indices implies that all the possible candidates are groups of I23, I2_1_3, Im3, I432, and I43m symmetry. For other body-centered I-type crystals suffer some systematic absence of various planes. In detail, Ia3 lacks 2, I4_1_32 does 4, I43d does 2 and 4, and Ia3d does 2, 4, 10, 12, etc. The periodicity, or equivalently the lateral unit cell length *c* is obtained as $c=2\pi /(q_{1}/\sqrt{2})$ = $6R_{G}$, which is exactly identical to our visual inspection of the morphology. 

It is observed from a careful inspection that the new morphology suffers missing mirror plane along y-axis. We then intuitively consider Im3 as a strong candidate. For the further identification of the newly found morphology as possessing Im3 symmetry, we try to check its known equivalent Wyckoff positions. Table 2 lists the first three Wyckoff positions along with their discretized coordinates and the local A block density $\eta_{A}(\vec{r})$. It is seen that there is the near perfect equivalence of $\eta_{A}(\vec{r})$ for the first two position groups. Even though we observe the dual way of equivalence with half-filled and half-depleted positions for the third position group, it is deduced that the given peculiar morphology should belong to the crystals of Im3 symmetry. 

**Figure 3:**
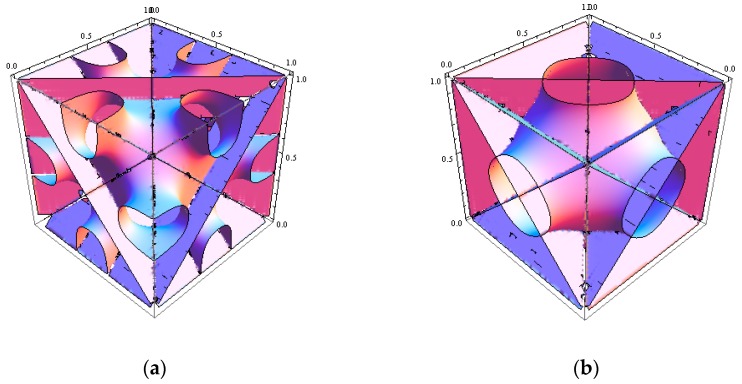
Contour plots for generic surface equations for Im3 mesophase using (**a**) (132) reflection and (**b**) (130) reflection along with their equivalent ones.

**Figure 4:**
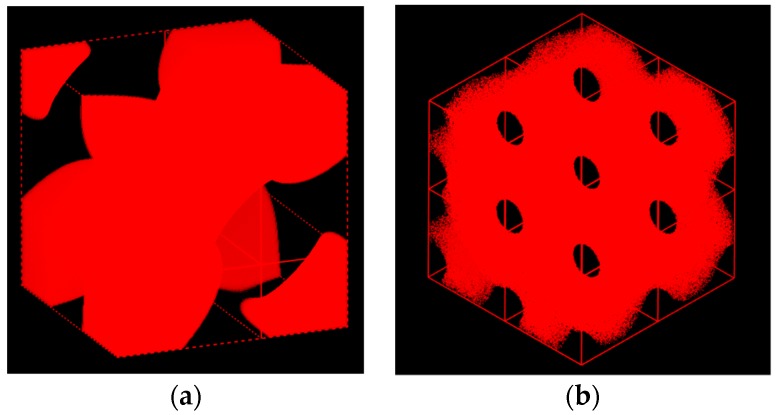
Three-dimensional morphology of the Im3 evolved from the generic test reflection given in Equation (8). Its unit cell morphology is given in plot (**a**) and its expansion in a box of 10 × 10 × 10$R_{G}^{3}$ is shown in plot (**b**). A domain is represented by red color and B domain as the matrix is intentionally removed.

In order to attest to the new morphology having Im3 symmetry, let us attempt to start with a generic test reflections for Im3 studied by Germer [40]. In his thesis, there are eight such cases to play with. The reflections with (*hkl*) = (130) and (*hkl*) = (132) are the first two to be taken. For simplification purposes, we turned off finite compressibility and took AB diblock copolymer with $\phi_{A}=0.4$ at Nχ=14. Our trial with (132) reflection is given as:

(11) $$\begin{array}{l} \psi (\vec{r})=\psi_{n}[\cos[6\pi x]\cos[4\pi y]\cos[2\pi z]+\cos[2\pi x]\cos[6\pi y]\cos[4\pi z]\\ +\cos[4\pi x]\cos[2\pi y]\cos[6\pi z]-(\cos[4\pi x]\cos[6\pi y]\cos[2\pi z]\\ +\cos[2\pi x]\cos[4\pi y]\cos[6\pi z]+\cos[6\pi x]\cos[2\pi y]\cos[4\pi z])] \end{array}$$

where $\psi_{n}$ is a proper amplitude and the local composition for A block is taken as $\phi_{A}(\vec{r})=\phi_{A}+\psi (\vec{r})$. The contour plot for the generic surface in Equation (11) is shown in Figure 3a. The SCFT simulations for the copolymer melts were undertaken in a tentative periodic cubic box of 5 × 5 × 5$R_{G}^{3}$, considering the simulation box should be smaller than that in the compressible situation. The simulation box was discretized into 32^3^ lattice cells, and each chain contour was discretized into 40 + 60 segments for A and B blocks, respectively. The modified diffusion equation in Equation (3) along with Equations (4–6) were solved via the pseudospectral scheme [39]. The iteration at a given condition was continued until the incompressibility constraint (=$\sum \phi_{i}-1$) was less than $2.5\times 10^{-7}$. In the same computational environment, it took ~13 min per 1k iterations for incompressible AB copolymer melts, and about 8.5k iterations were necessary in this case to reach the target tolerance. As was seen in Figure 4, the simulation was found to yield exactly the same morphology given in Figure 1 that we identify as *Im3* symmetry for the ABC copolymer. The periodicity or the lateral unit cell length *c* was obtained as $c=2\pi /(q_{1}/\sqrt{2})$ = $5R_{G}$, as it should. The correlation function $S_{AA}(q)$ for A block is given in Figure S1 as a Supplementary Material. It needs to be mentioned that our second trial with the initial density field generated using (130) reflection, whose contour plot is shown in Figure 3b, turns out that the evolved morphology was merely hexagonal P6/mm cylinders. 

### 3.2. Metatron’s Cube with Pn3m Symmetry

**Figure 5:**
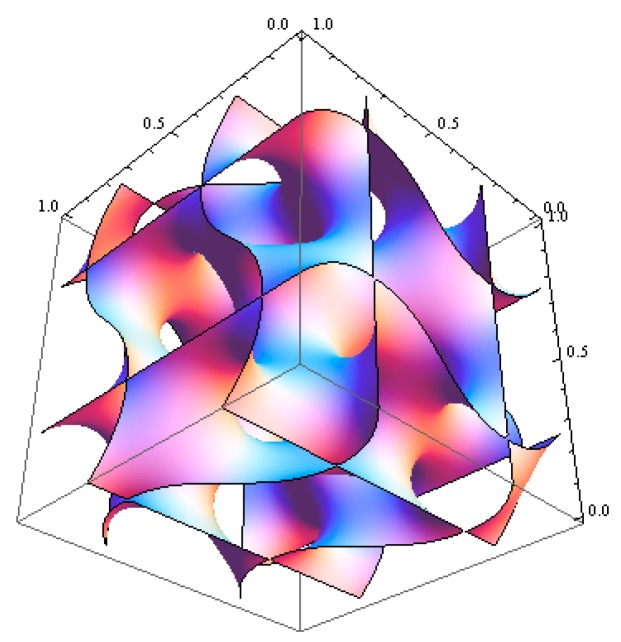
Contour plot of the generic C(±Y) surface given in Equation (12).

Our second concern is to start with known minimal surfaces to yield the corresponding morphologies, as we were successful in generating for the first time I43d structure in molten AB block copolymers in our previous report. There is such a surface named C(±Y) [41], which contains the reflections from (111) plane and (210) plane with its equivalent ones as:

(12) $$\begin{array}{ll} \psi (\vec{r})=-2 & \cos[2\pi x]\cos[2\pi y]\cos[2\pi z]+\\ & \sin[4\pi x]\sin[2\pi y]+\sin[4\pi y]\sin[2\pi z]+\sin[4\pi z]\sin[2\pi x]=0 \end{array}$$

whose generic shape is depicted in Figure 5 as follows.

Those reflections are the first two of a crystal with Pa3 symmetry group. Then, the SCFT simulations for the copolymer melts were undertaken in a tentative periodic box of 5 × 5 × 5$R_{G}^{3}$. The simulation box was discretized into 32^3^ lattice cells, and each chain contour was discretized into 40 + 60 segments. Finite compressibility was turned off. The modified diffusion equation was solved and the iteration at a given condition was continued until the target function (=$\sum \phi_{i}-1$) was less than $2.5\times 10^{-7}$. In the same computational environment, less than 5k iterations were necessary to reach the target tolerance.

**Figure 6:**
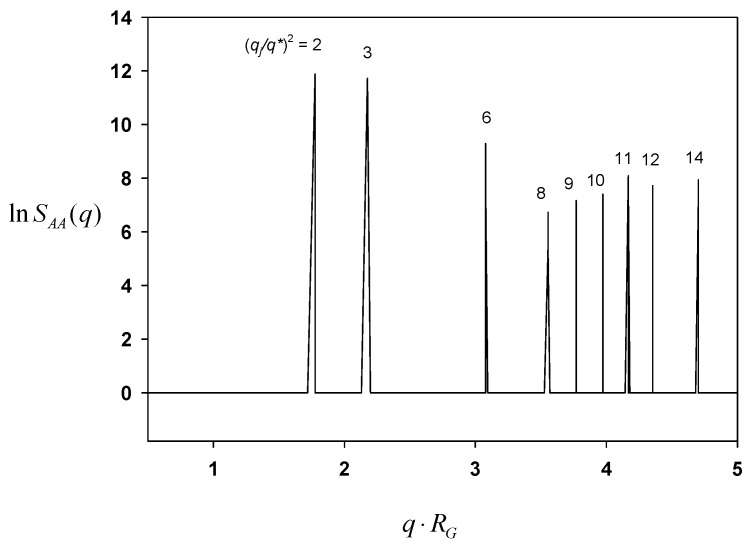
Correlation function $S_{AA}(q)$ for A block plotted against the scattering vector *q* for the morphology evolved from Equation (12).

In order to elucidate the symmetry of the resultant morphology, again the Fourier transform of a second-order correlation function was calculated. Figure 6 shows $S_{AA}(q)$ for A block as a function of the dimensionless scattering vector $q\cdot R_{G}$. There are a series of characteristic peaks at $q\cdot R_{G}$ = 1.7772, 2.1766, 3.0781, 3.5543, 3.7699, 3.9738, 4.1678, etc. The calculation of $2{\left(q/q_{1}\right)}^{2}$, where $q\cdot R_{G}$ = 1.7772, exhibits integers as 2, 3, 6, 8, 9, 10, 11, etc. It is perceived that all such (*hkl*) indices point to *Pn3m* symmetry. The periodicity or the lateral unit cell length *c* is obtained as $c=2\pi /(q_{1}/\sqrt{2})$ = $5R_{G}$, which is exactly what is observed in the morphology. 

**Figure 7:**
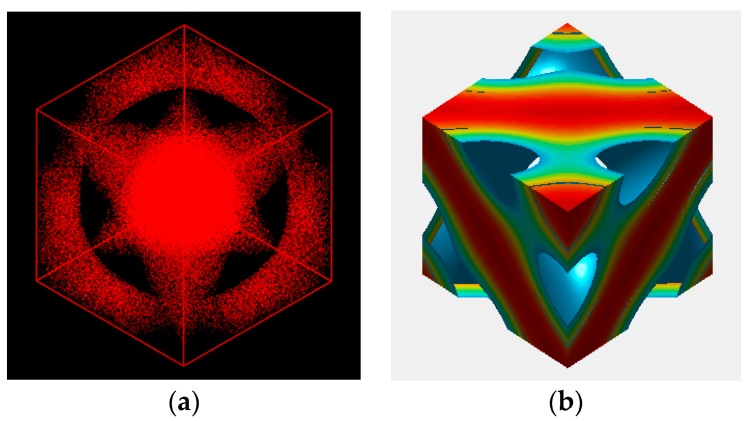
Three-dimensional morphology of the mesophase evolved from the generic test reflection given in Equation (12) in a simulation box (unit cell) of 5 × 5 × 5$R_{G}^{3}$. Its image is depicted by using Mesodyn (**a**) and also by Matlab (**b**) just for comparison purposes. A domain as the dispersed phase is only drawn here.

Using Mesodyn and also Matlab for comparison purposes, we tried to visualize the 3-dimensional morphology of the copolymer melt. While the well-known double diamond structure with Pn3m symmetry is expected to appear, Figure 7 portrays a totally new image of the morphology for the copolymer. Inside the cube, two triangular A-domains are connected with one of them rotated by 180 degrees to form a bicontinuous morphology. The B-domains then run through the triangular A-domains via three channels. The overall shape of the new morphology is considered to resemble the sacred Metatron’s cube [42]. It is noted that the channels are connected with tetrapod units. 

For the further identification of the newly found morphology as possessing Pn3m symmetry, we try to check its known equivalent Wyckoff positions. Table 3 lists the first three Wyckoff positions along with their discretized coordinates and the local A block composition $\phi_{A}(\vec{r})$. As the overall variations in $\phi_{A}(\vec{r})$ for each position groups are small, it is legitimate to claim that the given peculiar morphology belongs to the crystals of Pn3m symmetry.

### 3.3. P432 Symmetry

Our third concern is to start with the same C(±Y) surface, but the SCFT simulations for the copolymer melts are undertaken in a tentative periodic box of 10 × 10 × 10$R_{G}^{3}$. The simulation box was again discretized into 32^3^ lattice cells, and each chain contour was discretized into 40 + 60 segments for A and B blocks, respectively. Finite compressibility was again turned off. The iteration at a given condition was continued until the target function (=$\sum \phi_{i}-1$) was less than $2.5\times 10^{-7}$. In this case, 20k iterations were necessary to attain the target accuracy.

**Figure 8:**
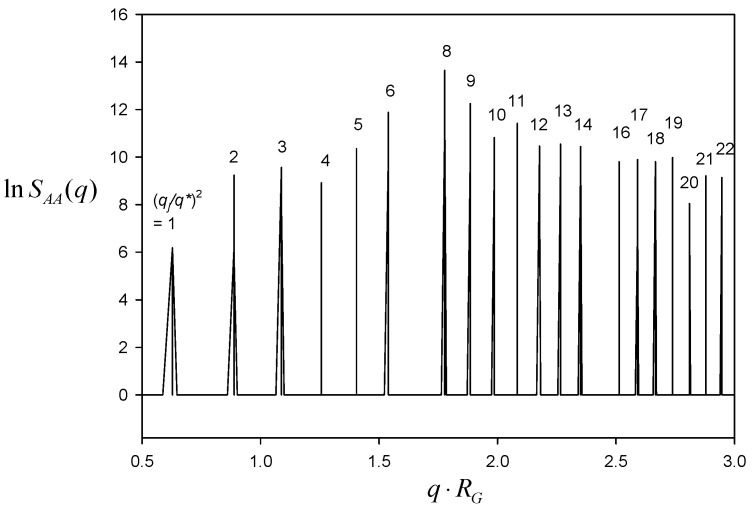
Correlation function $S_{AA}(q)$ for A block plotted against the scattering vector *q* for the morphology evolved from C(±Y) surface in a periodic box of 10 × 10 × 10$R_{G}^{3}$.

Figure 8 depicts $S_{AA}(q)$ for A block as a function of $q\cdot R_{G}$. The characteristic peaks turn up at $q\cdot R_{G}$ = 0.6283, 0.8886, 1.0883, 1.2566, 1.4050, 1.5391, 1.7772, etc. The calculation of ${\left(q/q_{1}\right)}^{2}$, where $q_{1}\cdot R_{G}$ = 0.6283, literally exhibits all possible integers as 1, 2, 3, 4, 5, 6, 8, etc. The crystals with all possible (*hkl*) indices can be some primitive groups of P23, Pm3, P432, P43m, and Pm3m symmetry. The periodicity or the lateral unit cell length *c* was obtained as $c=2\pi /q_{1}$ = $10R_{G}$, as it should. 

**Figure 9:**
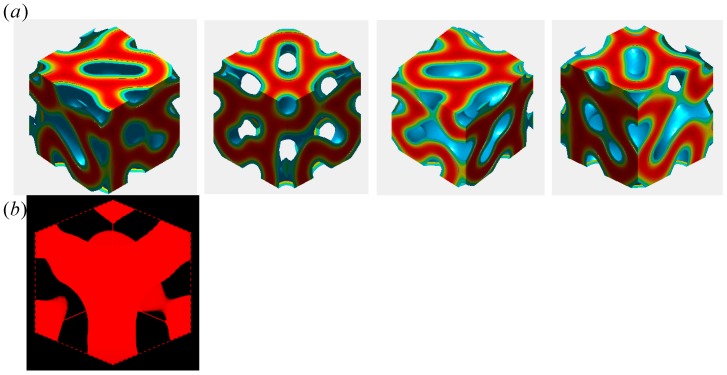
Three-dimensional morphology of the network mesophase evolved from C(±Y) surface in a periodic box of 10 × 10 × 10$R_{G}^{3}$. The unit cell morphology is depicted in plot (**a**) by using Matlab in four different angles and its 1/8th piece is shown in plot (**b**) by using Mesodyn just to reveal the tripod connections of the channels. A domain as the dispersed phase is only drawn here.

Using Matlab and Mesodyn, we provide the visualization of the 3-dimensional morphology for the copolymer melt. Figure 9a portrays another new morphology for the copolymer. It is seen from its 3-dimensional view that the B domains form a bicontinuous network with the wholly connected channels over the entire system to make many holes. The new morphology is considered to have no mirror planes along any axes. This notion rules out Pm3, P43m, and Pm3m symmetry, and there remain P23 and P432. It is then natural for us to assign P432 symmetry to this new mesophase, since P432 is a supergroup of P23. Furthermore, it is noted that the structural unit is a tripod, as is seen in the one-eighth piece of the unit cell in Figure 9b. 

For the further identification of this morphology as possessing P432 symmetry, we try to check its known equivalent Wyckoff positions. Table 4 lists the first four Wyckoff positions along with their discretized coordinates and the local A block composition $\phi_{A}(\vec{r})$. As there is a satisfactory equivalence of $\phi_{A}(\vec{r})$ for the first four position groups, it is considered that the given peculiar morphology belongs to the crystals of P432 symmetry.

### 3.4. Equilibrium Periodicity and Free Energies in the Incompressible Picture

Up to now, we have identified three new morphologies with Im3, Pn3m, and P432 crystal symmetry. It is necessary to compare the free energies of the newly evolved nanostructures with those of previously known structures such as double gyroids and others. For this purpose, the chain architecture was fixed to the linear AB diblock copolymer and the compositions were set to $\phi_{A}$ = 0.4. Finite compressibility was turned off and the effective Flory–Huggins χ is set to χ = 14/*N*. Then, the simulation box sizes, which are commensurable to the unit cells of the given structures, are to be optimized. This procedure is necessary for a fair comparison of the relative stability and accessibility of given morphologies. Table 5 lists the three new morphologies along with all the known morphologies including double gyroids, Fddd, P6/mm cylinders, Im3m BCC spheres, and Pn3m double diamonds. We also added in this table the recently identified holey morphologies in our previous work such as I43d morphology of genus 21 and Ia3d morphology of genus 25 [22]. Single gyroids with I4_1_32 symmetry are also included. It is well known that double gyroids are the stable morphology at the given segregation level and composition.

It is seen from Table 5 that Im3 structure reveals the optimized unit cell of ${\left(5.007R_{G}\right)}^{3}$. Its free energy is slightly greater than that of Fddd, and lower than that of P6/mm cylinders. The morphology with P432 possesses a bigger unit cell of ${\left(10.131R_{G}\right)}^{3}$ than double gyroids. Its free energy is slightly greater than that of P6/mm cylinders and that of Lamellae. It is followed by Metatron’s cube with Pn3m symmetry in its unit cell of ${\left(5.304R_{G}\right)}^{3}$, where its free energy is almost the same as that of conventional BCC Im3m spheres in an almost identical unit cell. BCC spheres are followed by double diamonds with Pn3m symmetry. The optimized free energies of the remaining morphologies lie in between those of double diamonds and disorder.

At the present conditions of chain architecture, compositions, and segregation strength, the three new morphologies are metastable. However, it was reported by Matsen [43,44] and later by Escobedo et al. [45,46,47] that double diamonds can be stabilized over double gyroids by blending with the homopolymers of the minor component through the relief of packing frustration. Shi and co-workers also suggested a different tactic to stabilize complex morphologies by blending two AB diblock copolymers of different sizes, or in other words making the bidisperse blends of AB copolymers [48]. It is therefore the topic of our future study to pursue such blending technique to find the condition where our new morphologies can be stabilized, especially targeting where double diamonds are stable. It is necessary to perform an extensive and thorough investigation into the vastly extended parameter space including the ratio of homopolymer size to copolymer size and homopolymer compositions.

As the present work employs the SCFT, our results are subject to the known limitation of the mean-field theory. Nonetheless, SCFT theories including ours have been the most successful with their predictability of phase behaviors of inhomogeneous polymeric mixtures among various theories. It has been known that the Ginzburg–Landau region [33], where the mean-field theory breaks down, scales as $N^{-1/3}$ for block copolymers [49]. The concentration fluctuations may then alter the phase boundary for the copolymers of finite sizes. The fluctuation effects are allowed in some sophisticated analyses such as the Gaussian fluctuation method [33,50] or one-loop correction to the mean-field approach [51], which can be another topic of future studies regarding the stability of newly identified morphologies.

## 4. Conclusions

Here, we theoretically study triply periodic nanoscale mesophases of molten block copolymers in search of useful nanomaterials for catalytic activity or mass transport capability. Taking linear AB or ABC block copolymers as a model system, whose j-blocks are *N_j_*-mers with *N* being the overall size of the chosen copolymers, field-theoretic simulations based on Edwards Gaussian random-walk approach are performed for our purposes. Without finite compressibility, Helfand’s conventional self-consistent field analysis is undertaken to evaluate the canonical partition function at its saddle point while ensuring the incompressibility constraint. In case of compressible copolymers, the recently developed analysis is undertaken to combine Helfand’s theory with a molecular equation-of-state model.

It is firstly revealed for the compressible ABC copolymer with $\left(N_{A},N_{B},N_{C}\right)$ = (110,55,55) at the segregation level of $\left(N\chi_{AB},N\chi_{AC},N\chi_{BC}\right)$ = (16.3,16.13,16.77) that there evolves a new mesophase having Im3 symmetry, which loses a mirror reflection compared with that having Im3m symmetry. Then, a generic surface equation to include (132) reflection is used to evolve the identical Im3¯ mesophase for the incompressible AB diblock copolymer with $\phi_{A}=0.4$ at Nχ=14. It is secondly shown for the same AB copolymer that a minimal surface named C(±Y) is used to develop a new bicontinuous mesophase with a unit cell of ~${\left(5R_{G}^{}\right)}^{3}$ possessing *Pn3m* symmetry. Its channels exhibit the tetrapod connections. It is thirdly shown for the same copolymer that starting with a unit cell of ~${\left(10R_{G}^{}\right)}^{3}$ leads to the evolution of a totally different bicontinuous mesophase possessing P432 symmetry. This third morphology reveals the entirely connected channels of the domains of the minor component with tripod units. For the identification of these nanostructures, we employed the correlation (scattering) functions and 3-dimensional visualization along with checking their first appearing Wyckoff positions. It is further shown in case of incompressible AB copolymer with $\phi_{A}=0.4$ at Nχ=14 that the three new mesophases in their optimized unit cells have the free energies lying between those of stable double gyroids and metastable double diamonds. Those results urge the necessity to exert efforts on stabilizing them through a technique to relieve packing frustration such as blending.

## Figures and Tables

**Table 1:** Sets of molecular parameters of A/B/C constituents composing ABC triblock copolymers based on Cho–Sanchez model.

	Parameter	${\bar{\varepsilon }}_{jj}/k$ (K)	$\sigma_{j}$ (Å)	$N_{j}/M_{j}\cdot \pi \sigma_{j}^{3}/6$ (cm^3^/g)
Polymer				
A		4107	4.04	0.41857
B		3000		
C		3000		

**Table 2:** Wyckoff positions for Im3 morphology.

Multiplicity	Position	Discretized Coordinates	$\eta_{A}(\vec{r})$
2a	0,0,0	1,1,1	0.012331
	1/2,1/2,1/2	9,9,9	0.012329
6b	0,1/2,1/2	1,9,9	0.34353
	1/2,0,1/2	9,1,9	0.343527
	1/2,1/2,0	9,9,1	0.329676
	1/2,0,0	9,1,1	0.343811
	0,1/2,0	1,91,	0.343831
	0,0,1/2	1,1,9	0.329386
8c	1/4,1/4,1/4	5,5,5	0.029919
	1/4,3/4,3/4	5,13,13	0.344344
	3/4,1/4,3/4	13,5,13	0.344256
	3/4,3/4,1/4	13,13,5	0.030894
	3/4,3/4,3/4	13,13,13	0.029839
	3/4,1/4,1/4	13,5,5	0.344491
	1/4,3/4,1/4	5,13,5	0.344419
	1/4,1/4,3/4	5,5,13	0.030805

**Table 3:** Wyckoff positions for Metatron’s cube morphology with Pn3m symmetry.

Multiplicity	Position	Discretized Coordinates	$\phi_{A}(\vec{r})$
2a	0,0,0	9,9,9	0.924754
	1/2,1/2,1/2	25,25,25	0.912959
4b	1/4,1/4,1/4	1,1,1	0.184598
	1/4,3/4,3/4	17,17,17	0.109947
	3/4,1/4,3/4	17,1,17	0.109947
	3/4,3/4,1/4	1,17,17	0.109947
4c	3/4,3/4,3/4	17,17,17	0.890423
	3/4,1/4,1/4	1,1,17	0.819492
	1/4,3/4,1/4	1,17,1	0.819492
	1/4,1/4,3/4	17,1,1	0.819492

**Table 4:** Wyckoff Positions for P432 morphology.

Multiplicity	Position	Discretized Coordinates	$\phi_{A}(\vec{r})$
1a	0,0,0	1,1,1	0.905336
1b	1/2,1/2,1/2	17,17,17	0.890529
3c	0,1/2,1/2	1,17,17	0.921184
	1/2,0,1/2	17,1,17	0.86979
	1/2,1/2,0	17,17,1	0.903127
3d	1/2,0,0	17,1,1	0.882721
	0,1/2,0	1,17,1	0.911596
	0,0,1/2	1,1,17	0.91643

**Table 5:** Comparison of the free energies for various morphologies of AB diblock copolymer melt at Nχ = 14 and at $\phi_{A}=0.4$.

Types of Copolymers	Morphology (Symmetry Group)	$c/R_{G}$	βA/n
AB	Double gyroids (*Ia3d*)	8.727	3.2334
	Fddd	4.055 × 8.136 × 14.404	3.2358
	Im3	5.007	3.2364
	*P6/mm* (HEX)	4.064 × 7.040	3.2370
	LAM	3.553	3.2377
	P432	10.131	3.2408
	Metatron’s cube (*Pn3m*)	5.304	3.2442
	BCC (*Im3m*)	5.303	3.2442
	Double diamonds (*Pn3m*)	5.475	3.2451
	Single gyroid (*I4_1_32*)	5.012	3.2461
	I43d	8.800	3.2470
	*Ia3d* of ***g*** = 25 *^b^*	8.910	3.2532
	Disorder	-	3.3600

*^a^* Fddd and P6/mm (HEX) morphologies require more than one lattice constant. Therefore, we included the optimized box dimensions in full for them. *^b^* *g* indicates the genus, which implies the number of independent holes on the dividing surface. It needs to be mentioned that double gyroids with the same *Ia3d* symmetry possess *g* = 5. *^c^* The target function for the incompressibility constraint ($\left|\sum \phi_{i}-1\right|$) is less than 2.5 × 10^−7^ for all the morphologies given in this table.

## Supplementary Materials

The following are available online at https://www.mdpi.com/2073-4360/11/6/1081/s1, Figure S1: Correlation function $S_{AA}(q)$ for A block plotted against the scattering vector *q* for incompressible AB diblock copolymer melt at $\phi_{A}=0.4$ and at Nχ = 14, whose morphology is evolved from Equation (11). Figure S2: Local compositions profiles for the same copolymer exhibiting the lamellar morphology.
