# Supplementary material for: Identification of Some New Triply Periodic Mesophases from Molten Block Copolymers
Source: Polymers (Basel). 2019 Jun 25;11(6):1081. doi: 10.3390/polym11061081 (PMC6631806; doi:10.3390/polym11061081)
Supplement: Supplementary file 1 [file polymers-11-01081-s001.pdf]

Supplementary Material

Identification of Some New Triply Periodic Mesophases from Molten Block Copolymers

Junhan Cho

Department of Polymer Science & Engineering and Center for Photofunctional Energy Materials, Dankook University, 152 Jukjeon-ro, Suji-gu, Yongin-si, Gyeonggi-do 16890, Korea; jhcho@dankook.ac.kr

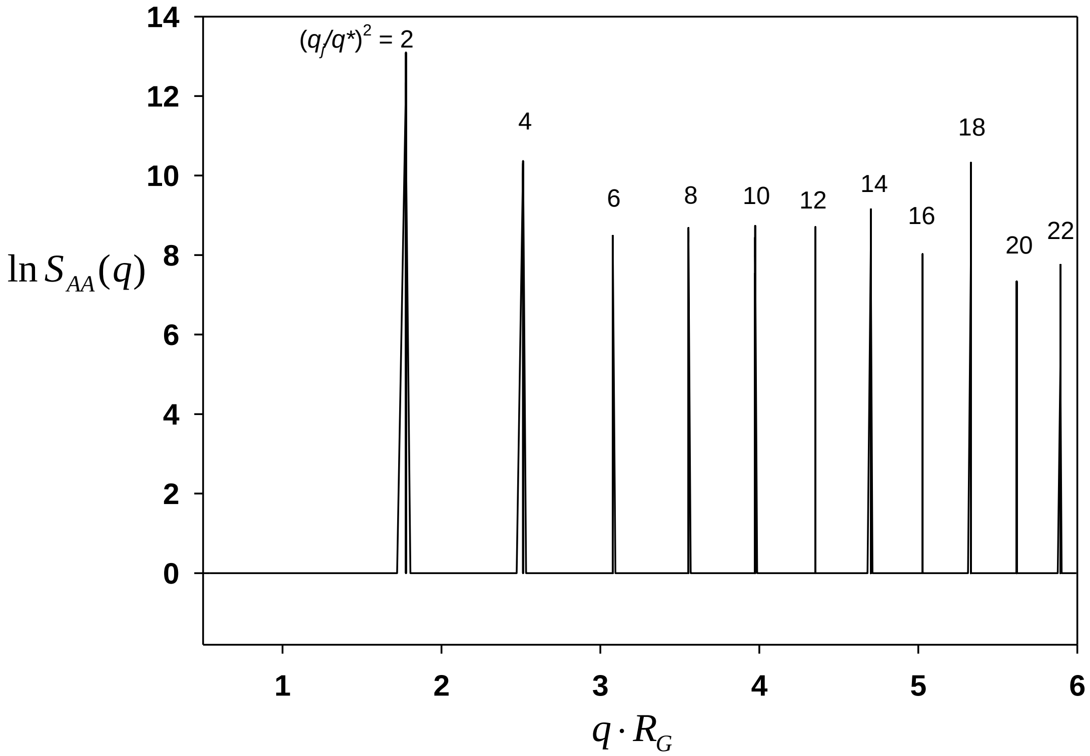

Figure S1. Correlation function $S_{AA}(q)$ for A block plotted against the scattering vector q for incompressible AB diblock copolymer melt at $\phi_A = 0.4$ and at $N\chi = 14$, whose morphology is evolved from Equation (11).

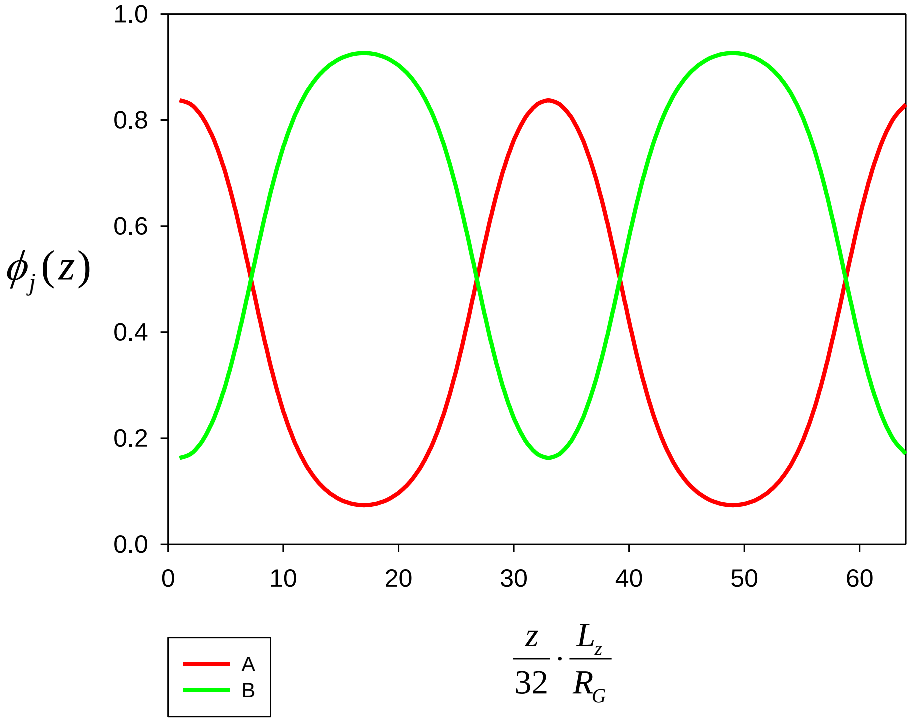

Figure S2. Local composition profiles for incompressible AB diblock copolymer melt at $\phi_A = 0.4$ and at $N\chi = 14$ exhibiting the lamellar morphology, where L_z indicates the equilibrium lamellar period.
